# Final Size for Epidemic Models with Asymptomatic Transmission

**DOI:** 10.1007/s11538-023-01159-y

**Published:** 2023-05-08

**Authors:** Carles Barril, Pierre-Alexandre Bliman, Sílvia Cuadrado

**Affiliations:** 1 Departament de Matemàtiques, Universitat Autònoma de Barcelona, Edifici C, Cerdanyola del Vallès, 08193 Barcelona, Spain; 2 Sorbonne Université, Inria, CNRS, Laboratoire Jacques-Louis Lions UMR7598, Equipe MAMBA, Université de Paris, 75005 Paris, France

**Keywords:** Final infection size, Symptomatic population, Reproduction number

## Abstract

The *final infection size* is defined as the total number of individuals that become infected throughout an epidemic. Despite its importance for predicting the fraction of the population that will end infected, it does not capture which part of the infected population will present symptoms. Knowing this information is relevant because it is related to the severity of the epidemics. The objective of this work is to give a formula for the total number of symptomatic cases throughout an epidemic. Specifically, we focus on different types of structured SIR epidemic models (in which infected individuals can possibly become symptomatic before recovering), and we compute the accumulated number of symptomatic cases when time goes to infinity using a probabilistic approach. The methodology behind the strategy we follow is relatively independent of the details of the model.

## Introduction

A main challenge in mathematical epidemiology is to be able to predict the evolution of a given epidemic depending on the measures taken. The final goal would be to find strategies that minimize the social and economic impact of the epidemic. The severity of an epidemic is assessed using different indicators. Among these indicators, there are two that are particularly important. One of them is the final size of the epidemic (for those infectious diseases that are not endemic), which gives the total number of individuals that will become infected during an epidemic. The other is the basic reproduction number (Diekmann et al. 1990), which gives the expected number of secondary infections produced by a typical infected individual at the beginning of the epidemic (when basically the entire population is susceptible and the infected population grows exponentially). It is clear that both indicators depend on the particularities of the infectious agent and the host population. Prevention and/or control measures seek to modify these particularities so that the epidemic is as mild as possible.

In certain epidemiological models, the two previous indicators are related in the sense that one can be deduced from the other. For instance, in the SIR model given by the ODE system

1 $$\begin{aligned} \left\{ \begin{array}{l} \dot{S}=-\beta S I, \\ \dot{I}=\beta S I-\gamma I,\\ S(0)=S_0,\quad I(0)=I_0, \end{array} \right. \end{aligned}$$

the basic reproduction number is $\mathcal {R}_0=\beta / \gamma$ and the fraction of infected population at the end of the epidemic, denoted by $\pi$, is the unique solution of the equation

2 $$\begin{aligned} \pi =1-e^{-\mathcal {R}_0\pi }. \end{aligned}$$

This type of relationship has been found in other, more elaborate models than system (1) (see, for instance, Ma and Earn 2006; Arino et al. 2007; Diekmann et al. 2013; Inaba 2014; Magal et al. 2016, 2018; Almeida et al. 2021). As suggested in Diekmann and Heesterbeek (2000) and explained in more detail in Miller (2012), for a relation of the form (2) to exist the infection probability from an individual to another must be independent of the moment in which the first individual becomes infected. The term “probability” in the above condition may suggest that this is a condition valid only for stochastic epidemiological models. The truth is, however, that it can be applied in deterministic models like system (1) simply by considering one of its possible underlying stochastic models (i.e. a stochastic process whose expected dynamics is explained by the deterministic system (1) when the total population is large enough).

When a significant portion of the infected population is asymptomatic, the impact of the epidemic that may be measured is more related to the number of individuals who will develop symptoms than to the final size of the infection. In these situations, therefore, it can be useful to study an indicator that gives the expected number of symptomatic cases that will occur throughout the epidemic, what we will call the final symptomatic size. Different models that distinguish between asymptomatic and symptomatic infected have been analysed, for instance, in Inaba and Nishiura (2008); Cushing and Diekmann (2016); Leung et al. (2018); Liu and Webb (2021); Barril et al. (2021b); Fitzgibbon et al. (2020). The aim of this article is to give analogous formulas to the relationship (1) in which the fraction of symptomatic population at the end of the epidemic (instead of the total fraction of infected) intervenes.

In Sect. 2 of this paper, the relationships between the basic reproduction number and the final symptomatic size are derived. In order to do this, we calculate the probability that a susceptible individual chosen at random at the beginning of the epidemic, what is called a test individual, ends up getting infected and showing symptoms. In order to introduce the ideas progressively, we distinguish three scenarios of increasing complexity: homogeneous populations (in which all individuals behave in the same way), populations where there is heterogeneity only among the susceptible, and populations where heterogeneity is present in both the susceptible and the infected individuals. Each of these scenarios is presented in its own subsection. In Sects. 3, 4 and 5, the results of Sect. 2 are applied to three examples covering the three possible scenarios. Specifically, in Sect. 3 we give an example of a homogeneous population in which the infected individuals are structured by their age of infection. We note that, despite the fact that the infected population is structured, the population is homogeneous since even though the behaviour of each infected individual varies throughout its life (it will be more or less infectious depending on the age of the infection), the way in which this behaviour varies is common to all infected individuals. Section 4 is devoted to an example of a heterogeneous population in which there are a finite number of different classes (typologies), both infected and susceptible. Section 5 generalizes this example by considering the susceptible and infected classes structured by a continuous variable.

## Does a Test Individual Present Symptoms?

As it was shown in Miller (2012) (and beforehand suggested in Diekmann and Heesterbeek 2000), the proportion of infected individuals in an outbreak can be computed as the probability that an individual chosen at random is infected at some instant during the epidemic. Under certain conditions on the population structure, this probability can be expressed in terms of $\mathcal {R}_0$, that is, *the expected number of new infections produced by an infected individual in a fully susceptible population*. A systematic procedure to compute $\mathcal {R}_0$ is based on the so-called next-generation operator, denoted by *G*, which gives the distribution of secondary infections as a function of the distribution of the primary infected individuals. It can be shown that $\mathcal {R}_0$ coincides with the spectral radius of *G* (see Diekmann et al. 1990; Inaba 2017; Barril et al. 2018).

In order to give a formula not for the size of the infected compartment, but for the proportion of individuals that have presented symptoms, it is enough to multiply the probability that a test individual is infected by the probability that this individual manifests the disease. In the following, we will discuss some general scenarios in which these two probabilities can be computed explicitly.

### Homogenous Population

Let us recall from Miller (2012) the derivation of the final infection size when all the individuals in the population are equivalent and the susceptibility level of an individual does not change during the epidemic (here by susceptibility level we mean the probability that a susceptible becomes infected per time unit and infected individual in the population). Notice that under this hypothesis, all susceptible individuals are equally likely to be infected by a random infected individual. That is, if we take a test individual from the susceptible population, then all infected individuals will infect this test individual with the same probability. On the contrary, if the susceptibility-level changes, then not all infected individuals will infect the test individual with the same probability. For example, if people change their social habits due to a high prevalence of the disease, then it will be more probable that the test individual gets the infection from an individual infected at the beginning of the epidemic (when people don’t adopt prevention measures) than from an individual infected later when the prevalence is high (when people adopt prevention measures). A simple ODE system in which the susceptibility level changes is:

$$\begin{aligned} \left\{ \begin{array}{l} S'(t)=-\frac{1}{N}\beta (I(t))S(t)I(t)\\ I'(t)=\frac{1}{N}\beta (I(t))S(t)I(t)-\gamma I(t) \end{array}\right. , \end{aligned}$$

where $\beta$ is a decreasing function and *N* denotes the total population. As far as we know, it is not possible to derive the final infection size of this kind of systems (when the susceptibility level changes in time) without integrating the trajectories of the system (it is possible, however, to derive bounds of the final infection size taking into account the maximum and minimum values that $\beta$ can take (Arino et al. 2007), and to derive analytical expressions for the final infection size in models in which the susceptibility level changes from one value to another permanently after the spread of the epidemics reaches some threshold (Gog and Hollingsworth 2021). This is why in this work we restrict ourselves to the case in which the susceptibility level of individuals is constant in time.

Let *K*(*N*) denote the number of individuals that will become infected during an epidemic in a population of *N* individuals. In general, *K*(*N*) is a random variable. Let us assume that

3 $$\begin{aligned} \lim _{N\rightarrow \infty } \frac{K(N)}{N}=\pi \in [0,1]\qquad \text {in probability}. \end{aligned}$$

The constant $\pi$, referred to as the *final infection size* from now on, represents the proportion of accumulated infected individuals at the end of the epidemics when the initial susceptible population is sufficiently large.

The previous assumption means that, for large population sizes, the random variable can be approximated by $\pi$. More precisely, that for all $\varepsilon >0$, the random variables *K*(*N*) satisfy

$$\begin{aligned} \lim _{N\rightarrow \infty }P\left( \left|\frac{K(N)}{N}-\pi \right|\ge \varepsilon \right) = 0. \end{aligned}$$

Under this hypothesis, it can be shown (as an application of the Poisson limit theorem) that

4 $$\begin{aligned} \lim _{N\rightarrow \infty } P\left( \text {Bin}\left( K(N),\frac{\lambda }{N}\right) =k\right) = P(\text {Poiss}(\lambda \pi )=k). \end{aligned}$$

where $\text {Bin}(n,p)$ denotes the binomial distribution with parameters $n\in \mathbb {N}$ and $p \in [0,1]$ and $\text {Poiss}(\lambda )$ denotes the Poisson distribution with parameter $\lambda >0$.

Since we are supposing that all infected individuals can infect a test individual *u* with the same probability, it follows that the probability that such a test individual becomes infected is

$$\begin{aligned} P \left( u \; \text {infected} \right) = P \left( \text {Bin} \left( K(N), \frac{\mathcal {R}_0}{N} \right) \geqslant 1 \right) = 1 - P \left( \text {Bin} \left( K(N), \frac{\mathcal {R}_0}{N} \right) = 0 \right) \end{aligned}$$

since each infected individual (from the total *K*(*N*)) can infect the test individual *u* with a probability $\mathcal {R}_0 / N$, where, as said before, $\mathcal {R}_0$ is the expected number of secondary infections that a primary infected individual produces when the population is fully susceptible. Then, in the limit $N \rightarrow \infty$, we have $P (u \text { infected}) = \pi$, and on the other hand due to assumption (3) via (4)

$$\begin{aligned} \lim _{N\rightarrow \infty } P \left( \text {Bin} \left( K(N), \frac{\mathcal {R}_0}{N} \right) = 0 \right) = P \left( \text {Poiss} \left( \pi \mathcal {R}_0 \right) = 0 \right) = e^{- \mathcal {R}_0 \pi }, \end{aligned}$$

so that

5 $$\begin{aligned} \pi = 1 - e^{- \mathcal {R}_0 \pi }. \end{aligned}$$

The previous formula for the final infection size can be used to derive the number of symptomatic cases that the epidemic will cause. Indeed, if $p_{\text {sym}}$ denotes the probability that an infected individual presents symptoms, then the probability that the test individual *u* presents symptoms is

6 $$\begin{aligned} \pi _{\text {sym}}:=p_{\text {sym}}P \left( u \; \text {infected} \right) =p_{\text {sym}}\pi . \end{aligned}$$

The probability $\pi _\text {sym}$ coincides with the proportion of symptomatic individuals at the end of the epidemic.

It can be shown that, if $\mathcal {R}_0>1$, Eq. (5) has a unique positive solution, which we denote by $\pi (\mathcal {R}_0)$. Moreover, $\pi (\mathcal {R}_0)$ is an increasing function of $\mathcal {R}_0$, which means that the larger $\mathcal {R}_0$ is, the larger the final infection size. As $\pi _{\text {sym}}$ is an increasing function of $\pi$, we also conclude that $\pi _\text {sym}$ is an increasing function of both $\mathcal {R}_0$ and $p_\text {sym}$.

### Heterogeneous Susceptible Population

In more realistic situations, the susceptibility is not the same for all individuals because there are physiological or behavioural differences between them. For instance, a probability of infection higher than average could be observed in immunosuppressed people (a physiological trait) or in promiscuous people (a behavioural trait). Epidemiological models capture this heterogeneity by structuring the population into compartments or classes, describing the population by a density function with respect to the structuring variable (Almeida et al. 2021; Inaba 2014; Lorenzi et al. 2021; Peng and Zhao 2012; Wang and Zhao 2012).

When the susceptibility level of individuals does not change during the epidemic and all infected individuals are equivalent, a generalization of (5) can be derived (Miller 2012). Structuring the susceptibility classes according to a variable *x*, we can define $\mathcal {R}_0 (x) > 0$ as the number of secondary infections an infected individual causes to susceptibles of type *x*. In particular, this definition implies that

$$\begin{aligned} \mathcal {R}_0 = \int \mathcal {R}_0 (x) \textrm{d} x. \end{aligned}$$

Let $s_0(x)$ be the normalized density of susceptibles within class *x* at the beginning of the epidemic (i.e. $s_0(x)N$ is the density of susceptibles of type *x* at that moment). Let *K*(*N*) be, as in Sect. 2.1, the number of individuals that will become infected during the epidemic in a population of *N* individuals. Then, using again (4), the probability that a test individual of type *x* becomes infected (for large *N*) can be expressed in terms of $\mathcal {R}_0(x)$ as:

$$\begin{aligned} \begin{aligned} P \left( u \; \text {infected} \vert u \text { is of type } x\right)&= \lim _{N\rightarrow \infty }P \left( \text {Bin} \left( K(N), \frac{\mathcal {R}_0 (x)}{s_0(x)N} \right) \geqslant 1 \right) \\&= \lim _{N\rightarrow \infty } 1 - P \left( \text {Bin} \left( K(N), \frac{\mathcal {R}_0 (x)}{s_0(x)N} \right) = 0 \right) \\&= 1 - P \left( \text {Poiss} \left( \frac{\mathcal {R}_0(x)}{s_0(x)} \pi \right) = 0 \right) = 1 - e^{- \frac{\mathcal {R}_0(x)}{s_0(x)} \pi }, \end{aligned} \end{aligned}$$

since each infected individual (from the total *K*(*N*)) can infect the test individual of type *x* with probability $\frac{\mathcal {R}_0 (x)}{s_0(x)N}$. Now we can define $\pi _s(x)$ so that $N \pi _s(x)$ gives the density of susceptible individuals of type *x* that will become infected at some point during the epidemic. Then, one has (interpreting $P(u \text { is of type }x)$ as the probability density of *u* being of type *x*)

7 $$\begin{aligned} \pi _s(x)=P \left( u \; \text {infected} \vert u \text { is of type }x\right) P(u \text { is of type }x) = \left( 1 - e^{- \frac{\mathcal {R}_0(x)}{s_0(x)}\pi }\right) s_0(x) \end{aligned}$$

and in particular

8 $$\begin{aligned} \pi = \int \pi _s (x) \textrm{d} x = \int \left( 1 - e^{- \frac{\mathcal {R}_0(x)}{s_0(x)}\pi }\right) s_0(x) \textrm{d} x = 1 - \int e^{- \frac{\mathcal {R}_0(x)}{s_0(x)}\pi }s_0(x) \textrm{d} x. \end{aligned}$$

where we have used that $\int s_0(x)=1$. This formula gives the final infection size of the epidemic. In order to know the final number of symptomatic cases, we have to introduce $p_\text {sym}(x)$ as the probability that a susceptible individual of type *x* presents symptoms after becoming infected, and then, the probability that a test individual of type *x* shows symptoms is

$$\begin{aligned} \pi ^\text {sym}_s(x):=p_\text {sym}(x) \pi _s(x). \end{aligned}$$

Since, from (7),

9 $$\begin{aligned} \pi ^\text {sym}_s(x)=p_\text {sym}(x)\left( 1 - e^{- \frac{\mathcal {R}_0(x)}{s_0(x)}\pi }\right) s_0(x), \end{aligned}$$

we obtain that the number of symptomatic cases that will be produced during the epidemic is:

10 $$\begin{aligned} \pi _\text {sym}=\int \pi ^\text {sym}_s(x)\textrm{d}x=\int p_\text {sym}(x)\left( 1 - e^{- \frac{\mathcal {R}_0(x)}{s_0(x)}\pi }\right) s_0(x) \textrm{d}x. \end{aligned}$$

Therefore, if $s_0(x)$, $\mathcal {R}_0(x)$ and $p_\text {sym}(x)$ are known, the fraction $\pi _\text {sym}$ can be obtained by solving first (8) in order to obtain $\pi$, and afterwards solving (10). Notice that, in addition to $\pi$ and $\pi _\text {sym}$, formulas (7) and (9) can be used to compute $\pi _s(x)$ and $\pi ^\text {sym}_s(x)$, which may give information on the susceptibles that are more vulnerable.

### Heterogeneous Susceptible and Infected Population

Let us now consider heterogeneity in both populations, susceptible and infected. As before, let $s_0(x)$ be the density of susceptibles within class *x* at the beginning of the epidemic (i.e. $s_0(x)N$ is the number of susceptibles of type *x* at that moment). Let us define $\mathcal {R}_0 (x,y) > 0$ as the number of secondary infections a primary infected individual of type *y* causes to susceptibles of type *x*. Notice that $\mathcal {R}_0 (x,y)$ depends on $s_0(x)$. Indeed, if there are no susceptibles of class *x* (i.e. if $s_0(x)=0$) then necessarily $\mathcal {R}_0(x,y)=0$.

Let us assume there is a finite number *n* of susceptible classes and a finite number *m* of infected classes. That is, if *x* and *y* denote the type of susceptible and infected individuals, respectively, then $x\in \{x_1,x_2,\dots ,x_n\}$ and $y\in \{y_1,y_2,\dots ,y_m\}$. The class a susceptible belongs to is fixed, in the sense that its class remains the same until it becomes infected. The same is assumed for infected individuals: the class an infected individual belongs to is always the same until it recovers. In analogy to *K*(*N*) in the previous subsections, let $K(N,y_i)$ be the accumulated infected individuals of type $y_i$ when the epidemic ends, and let us assume that

11 $$\begin{aligned} \lim _{N\rightarrow \infty } \frac{K(N,y_i)}{N}=\pi (y_i)\in [0,1]\qquad \text {in probability}. \end{aligned}$$

Here, $\pi (y_i)$ can be interpreted as the proportion of accumulated infected individuals of type $y_i$ at the end of the epidemic when *N* is large enough.

In this case, the probability that a test individual of type *x* becomes infected is (using property (4) once more)

$$\begin{aligned} \begin{aligned} P(u\text { infected } \vert&u \text { is of type } x)=\lim _{N\rightarrow \infty } 1-P(u\text { not infected } \vert u \text { is of type } x)\\&=\lim _{N\rightarrow \infty } 1-\prod _{i=1}^m P\left( \text {Bin}\left( K(N,y_i),\frac{\mathcal {R}_0(x,y_i)}{s_0(x)N}\right) =0\right) \\&= 1-\prod _{i=1}^m e^{-\frac{\mathcal {R}_0(x,y_i)}{s_0(x)}\pi (y_i)}=1-e^{-\sum _{i=1}^m \frac{\mathcal {R}_0(x,y_i)}{s_0(x)}\pi (y_i)}. \end{aligned} \end{aligned}$$

In particular, if whenever a susceptible individual of type $x_j$ is infected, it becomes an infected individual of type $y_j$ (that is, if there is a one-to-one correspondence between classes of susceptible and infected, which implies $m=n$), one then has

12 $$\begin{aligned} \begin{aligned} \pi (y_j)=\pi _s(x_j):=&P(u \text { infected } \vert u \text { is of type }x_j)P(u\text { is of type }x_j)\\ =&\left( 1-e^{-\sum _{i=1}^n \frac{\mathcal {R}_0(x_j,y_i)}{s_0(x_j)}\pi (y_i)}\right) s_0(x_j), \end{aligned} \end{aligned}$$

where $\pi _s(x_j)$ represents the fraction of susceptible individuals of class $x_j$ infected during the epidemic.

More generally, if whenever a susceptible individual of type $x_j$ is infected, it becomes an infected individual of type $y_k$ with probability $p_{x_j\rightarrow y_k}$ we have, for all $k\in \{1,\dots ,m\}$,

13 $$\begin{aligned} \begin{aligned} \pi (y_k)&=\sum _{j=1}^n P(u \text { infected } \vert u \text { is of type }x_j)P(u\text { is of type }x_j)p_{x_j\rightarrow y_k}\\&=\sum _{j=1}^n \left( 1-e^{-\sum _{i=1}^m \frac{\mathcal {R}_0(x_j,y_i)}{s_0(x_j)}\pi (y_i)}\right) s_0(x_j)p_{x_j\rightarrow y_k}, \end{aligned} \end{aligned}$$

and in this case $\pi _s(x_j)$, for $j\in \{1,\dots ,n\}$, can be expressed in terms of $\pi (y_k)$ as

$$\begin{aligned} \begin{aligned} \pi _s(x_j)&=P(u \text { infected } \vert u\text { is of type }x_j)P(u\text { is of type }x_j)\\ {}&=\left( 1-e^{-\sum _{i=1}^m \frac{\mathcal {R}_0(x_j,y_i)}{s_0(x_j)}\pi (y_i)}\right) s_0(x_j). \end{aligned} \end{aligned}$$

Let us note that, for both the particular case (12) and the general case (13), in order to find the vector $\pmb {\pi }:=(\pi (y_1),\dots ,\pi (y_m))$ we have to solve an equation of the form

$$\begin{aligned} \pmb {\pi }=F(\pmb {\pi }) \end{aligned}$$

with $F:\mathbb {R}^m\rightarrow \mathbb {R}^m$, where the image of *F* corresponds to the right-hand side of equations (13) for the different values of *k*, i.e. for $w\in \mathbb {R}^m$ and $k\in \{1,\dots ,m\}$, $F_k(w)$ is defined by:

14 $$\begin{aligned} F_k(w)=\sum _{j=1}^n \left( 1-e^{-\sum _{i=1}^m \frac{\mathcal {R}_0(x_j,y_i)}{s_0(x_j)}w_i}\right) s_0(x_j)p_{x_j\rightarrow y_k}. \end{aligned}$$

If *F* has a unique fixed point $\pmb {\pi }$ with nonnegative entries and the sequence of iterates $F^l(\pmb {\pi }_0)\rightarrow \pmb {\pi }$ when $l\rightarrow \infty$ for all $\pmb {\pi }_0$ with positive entries, then (13), interpreted as a fixed point equation, gives a method to obtain the infection final size vector. The following result guarantees exactly that.

#### Theorem 1

Let $\mathcal {R}_0$ be the reproduction number associated with the model considered. Then,

- if $\mathcal {R}_0<1$, then 0 is the only fixed point of *F* in the positive cone and $F^l(w)\rightarrow 0$ when $l\rightarrow \infty$ for all *w* in the positive cone,
- if $\mathcal {R}_0>1$, then there exists a fixed point $\pmb {\pi }\ne 0$ of *F* in the positive cone such that $F^l(w)\rightarrow \pmb {\pi }$ when $l\rightarrow \infty$ for all *w* satisfying
  $$\begin{aligned} w_i \ge \sum _{k=1}^n s_0(x_k) p_{x_k\rightarrow y_i}\qquad \forall i\in \{1,\dots ,m\}. \end{aligned}$$
  If $\pmb {\pi }$ is the only nonzero fixed point of *F* in the positive cone, then $F^l(w)\rightarrow \pmb {\pi }$ when $l\rightarrow \infty$ for all $w\ne 0$ of the positive cone.

#### Proof

See Appendix. $$\square $$

Generically, when $\mathcal {R}_0>1$, *F* has only one nonzero fixed point in the positive cone. The degenerate cases with multiple nonzero fixed points correspond to scenarios in which the infection may not be able to spread to all infected classes. This occurs, for instance, if the secondary cases caused by an infected individual are always of the same class as the primary infection. In this case, there are multiple final infection sizes depending on the initial distribution of infected individuals. From now on, we assume that *F* has only one nonzero fixed point.

The vector $\pmb {\pi }=(\pi (y_1),\dots ,\pi (y_m))$ then gives the fraction of infected individuals of different types at the end of the epidemic and allows to compute the proportion of infected individuals in the population at the end of the epidemic, since

15 $$\begin{aligned} \pi =\sum _{k=1}^m \pi (y_k). \end{aligned}$$

Let us note that

$$\begin{aligned} \begin{aligned} \sum _{k=1}^m \pi (y_k)&= \sum _{k=1}^m \sum _{j=1}^n \left( 1-e^{-\sum _{i=1}^m \frac{\mathcal {R}_0(x_j,y_i)}{s_0(x_j)}\pi (y_i)}\right) s_0(x_j)p_{x_j\rightarrow y_k} \\&= \sum _{j=1}^n \left( 1-e^{-\sum _{i=1}^m \frac{\mathcal {R}_0(x_j,y_i)}{s_0(x_j)}\pi (y_i)}\right) s_0(x_j) \sum _{k=1}^m p_{x_j\rightarrow y_k} \\&= \sum _{j=1}^n \left( 1-e^{-\sum _{i=1}^m \frac{\mathcal {R}_0(x_j,y_i)}{s_0(x_j)}\pi (y_i)}\right) s_0(x_j) = \sum _{j=1}^n \pi _s(x_j) \end{aligned} \end{aligned}$$

as expected since the sum of infected individuals must coincide with the sum of all susceptible individuals that became infected.

The previous arguments cannot be applied when the set of infected classes is not finite. If the possible infected classes form an open set of an Euclidean space, denoted by $\Omega _Y$, then *K*(*N*, *y*) could be 0 almost surely for all $y\in \Omega _Y$. To address this problem, instead of *K*(*N*, *y*) one must consider $K(N,\omega )$ defined as the accumulated number of infected individuals of types $y\in \omega \subset \Omega _Y$ at the end of the epidemics (where $\omega$ is a Lebesgue measurable set), and assumption (11) should be replaced by

$$\begin{aligned} \lim _{N\rightarrow \infty } \frac{K(N,\omega )}{N} = \pi (\omega )\in [0,1] \end{aligned}$$

in probability for all Lebesgue-measurable $\omega \subset \Omega _Y$.

In this case, $\pi (\omega )$ corresponds to the proportion of accumulated infected individuals with types $y\in \omega$ at the end of the epidemic when *N* is large enough.

From now on, let us restrict ourselves to models in which the function $\pi$ can be written in terms of an integrable function $\tilde{\pi }:\Omega _Y\rightarrow \mathbb {R}$ as

$$\begin{aligned} \pi (\omega )=\int _\omega \tilde{\pi }(y)\textrm{d}y. \end{aligned}$$

Notice that if $\Omega _Y=[0,1]$, such a function $\tilde{\pi }$ exists provided the mapping $y\mapsto \pi ([0,y])$ (which is increasing) is continuous. The fraction of infected population at the end of the epidemic when *N* tends to infinity (i.e. the *final infection size*) is

$$\begin{aligned} \pi =\lim _{N\rightarrow \infty }\frac{K(N,\Omega _Y)}{N}= \pi (\Omega _Y)= \int _{\Omega _Y} \tilde{\pi }(y) \textrm{d}y. \end{aligned}$$

Consider now a random variable *Y* with density

16 $$\begin{aligned} f_Y(y)=\frac{\tilde{\pi }(y)}{\pi }. \end{aligned}$$

Then, since the accumulated number of infected individuals when the epidemic ends is $K(N,\Omega _Y)=:K(N)$, it follows

17 $$\begin{aligned} P(u \text { infected } \vert u \text { is of type }x)= & {} 1-P(u \text { not infected } \vert u \text { is of type }x)\nonumber \\= & {} \lim _{N \rightarrow \infty } \left( 1-\prod _{i=1}^{K(N)} \left( 1-\frac{\mathcal {R}_0(x,Y_i)}{s_0(x)N}\right) \right) \end{aligned}$$

for all $x\in \Omega _X$, where $\Omega _X$ is the set of all susceptible classes, $s_0(x)$ is the initial distribution of susceptibles and $Y_i$ are independent random variables with density given by (16). Notice that, when *x* takes values in a continuum, then both $s_0(x)N$ and $\mathcal {R}_0(x,y)$ could be densities of individuals with respect to the susceptible structuring variable *x* (and not individuals as in the case of a finite number of susceptible classes).

In order to rewrite the right-hand side of (17) in terms of $\tilde{\pi }$, notice that

$$\begin{aligned}&\lim _{N\rightarrow \infty } \prod _{i=1}^{K(N)} \left( 1-\frac{\mathcal {R}_0(x,Y_i)}{s_0(x)N}\right) = \exp \left( \lim _{N\rightarrow \infty }\sum _{i=1}^{K(N)} \log \left( 1-\frac{\mathcal {R}_0(x,Y_i)}{s_0(x)N}\right) \right) \\&\quad = \exp \left( \lim _{N\rightarrow \infty }\sum _{i=1}^{K(N)} \sum _{j=1}^\infty - \frac{1}{j}\left( \frac{\mathcal {R}_0(x,Y_i)}{s_0(x)N}\right) ^j\right) \\&\quad = \exp \left( \lim _{N\rightarrow \infty }\sum _{i=1}^{K(N)} \sum _{j=1}^\infty -\frac{1}{j}\left( \frac{\mathcal {R}_0(x,Y_i)}{s_0(x)K(N)}\right) ^j\left( \frac{K(N)}{N}\right) ^j\right) \\&\quad = \exp \left( -\frac{\mathbb {E}(\mathcal {R}_0(x,Y))}{s_0(x)}\pi +\lim _{N\rightarrow \infty }\sum _{i=1}^{K(N)}\sum _{j=2}^\infty -\frac{1}{j}\left( \frac{\mathcal {R}_0(x,Y_i)}{s_0(x)K(N)}\right) ^j\left( \frac{K(N)}{N}\right) ^j\right) . \end{aligned}$$

In particular, if the moments of $\mathcal {R}_0(x,Y)$ are finite (for all $x\in \Omega _X$), we have

$$\begin{aligned}&\lim _{N\rightarrow \infty }\sum _{i=1}^{K(N)}\sum _{j=2}^\infty -\frac{1}{j}\left( \frac{\mathcal {R}_0(x,Y_i)}{s_0(x)K(N)}\right) ^j\left( \frac{K(N)}{N}\right) ^j \\&\qquad =\lim _{N\rightarrow \infty }\sum _{j=2}^\infty -\frac{1}{j}\frac{\mathbb {E}(\mathcal {R}_0(x,Y)^j)}{s_0(x)^jK(N)^{j-1}}\left( \frac{K(N)}{N}\right) ^j=0, \end{aligned}$$

and

$$\begin{aligned} P(u \text { infected } \vert u \text { is of type }x)=1-e^{-\frac{\mathbb {E}(\mathcal {R}_0(x,Y))}{s_0(x)}\pi }=1-e^{-\int _{\Omega _Y} \frac{\mathcal {R}_0(x,y)}{s_0(x)}\tilde{\pi }(y)\textrm{d}y}. \end{aligned}$$

Therefore, with an argument analogous to the one used in the finite case, if a susceptible individual of type *x*, once infected, becomes an infected individual of type *y* with probability 1, we have (interpreting $P(u \text { is of type }x)$ as the probability density of *u* being of type *x*)

18 $$\begin{aligned} \begin{aligned} \tilde{\pi }(y)=\tilde{\pi }_s(x):=&P(u \text { infected } \vert u \text { is of type }x)P( u \text { is of type }x) \\ =&\left( 1-e^{-\int _{\Omega _Y} \frac{\mathcal {R}_0(x,y)}{s_0(x)}\tilde{\pi }(y)\textrm{d}y}\right) s_0(x). \end{aligned} \end{aligned}$$

In general, if $p_i(x,y)$ denotes the probability density that a susceptible individual of type *x*, once infected, becomes an infected individual of type *y*, then

19 $$\begin{aligned} \tilde{\pi }(y)=\int _{\Omega _X} \tilde{\pi }_s(x)p_i(x,y)\textrm{d}x=\int _{\Omega _X} \left( 1-e^{-\int _{\Omega _Y} \frac{\mathcal {R}_0(x,z)}{s_0(x)}\tilde{\pi }(z)\textrm{d}z}\right) s_0(x)p_i(x,y)\textrm{d}x.\nonumber \\ \end{aligned}$$

From this integral equation, one could, at least numerically, find $\tilde{\pi }(y)$ (and in particular the fraction $\pi =\int _{\Omega _Y}\tilde{\pi }(y)\textrm{d}y$ of infected individuals during the epidemic).

In order to be able to compute the fraction of symptomatic cases, one more ingredient should be introduced in the problem, meaning the probability that an infected individual of type *y* formerly susceptible of type *x* develops symptoms. Let $p_{\text {sym}}(x,y)$ denote this probability. Then, $\pi _s(x)p_i(x,y)p_{\text {sym}}(x,y)$ will be the distribution of symptomatic individuals with respect to the infected and susceptible types, and the total fraction of symptomatic individuals during the epidemic will be

20 $$\begin{aligned} \pi _{\text {sym}}=\int _{\Omega _Y\times \Omega _X} \tilde{\pi }_s(x)p_i(x,y)p_{\text {sym}}(x,y)\textrm{d}y\,\textrm{d}x. \end{aligned}$$

In the particular case that the probability of having symptoms only depends on the class to which the infected individual belongs, i.e., if $p_\text {sym}(x,y)=p_\text {sym}(y)$, then, using (19) the following identity holds for $\pi _{\text {sym}}$

21 $$\begin{aligned} \pi _{\text {sym}}=\int _{\Omega _Y} \tilde{\pi }(y)p_{\text {sym}}(y)\textrm{d}y. \end{aligned}$$

In the case of finite classes of both susceptible and infected individuals, formula (20) reduces to

$$\begin{aligned} \pi _{\text {sym}}=\sum _{i=1}^n\sum _{j=1}^m \pi _s(x_i)p_{x_i\rightarrow y_j}p_{\text {sym}}(x_i,y_j). \end{aligned}$$

In fact, one could define

$$\begin{aligned} \pi ^{\text {sym}}_s(x_i) = \sum _{j=1}^m \pi _s(x_i)p_{x_i\rightarrow y_j}p_{\text {sym}}(x_i,y_j)\qquad \forall i\in \{1,\dots ,n\} \end{aligned}$$

and

$$\begin{aligned} \pi _{\text {sym}}(y_j) = \sum _{i=1}^n \pi _s(x_i)p_{x_i\rightarrow y_j}p_{\text {sym}}(x_i,y_j)\qquad \forall j\in \{1,\dots ,m\}, \end{aligned}$$

in such a way that $\pi ^{\text {sym}}_s(x_i)$ is the fraction (with respect to the total population *N*) of susceptibles in class $x_i$ that will end up developing symptoms and $\pi _{\text {sym}}(y_j)$ is the fraction (also with respect to the total population *N*) of individuals that will end up showing symptoms and that during the asymptomatic phase were infected individuals of type $y_j$.

When the classes of susceptible and infected individuals are equivalent (that is, if $n=m$ and $p_{x_i\rightarrow y_j}=1$ if $i=j$ and 0 otherwise, implying $\pi _s(x_i)=\pi (y_i)$), one has

$$\begin{aligned} \pi ^{\text {sym}}_s(x_i) = \pi _{\text {sym}}(y_i). \end{aligned}$$

Moreover, in this case the probability of an infected individual developing symptoms does not depend on its type as susceptible (since this is already determined). Thus, defining $p_{\text {sym}}(y_i)=p_{\text {sym}}(x_i,y_i)$, formula (21) becomes

22 $$\begin{aligned} \pi _{\text {sym}}=\sum _{i=1}^n \pi _{\text {sym}}(y_i) = \sum _{i=1}^n \pi (y_i)p_{\text {sym}}(y_i). \end{aligned}$$

## Age of Infection Model

Let us consider the following age of infection structured model considering both asymptomatic and symptomatic individuals:

23 $$\begin{aligned} \begin{array}{l} S' (t) = \displaystyle - \frac{1}{N} \left( S (t) \int _0^T \beta _1 (\tau ) i (t, \tau ) \textrm{d} \tau + \beta _2 S (t) J (t) \right) ,\\ \partial _t i (t, \tau ) + \partial _{\tau } i (t, \tau ) = - \gamma _1 (\tau ) i (t, \tau ),\\ i (t, 0) = \displaystyle \frac{1}{N} \left( S (t) \int _0^T \beta _1 (\tau ) i (t, \tau ) \textrm{d} \tau + \beta _2 S (t) J (t) \right) ,\\ J' (t) = p i (t, T) - \gamma _2 J (t), \end{array} \end{aligned}$$

where the state variables *S*, *i* and *J* represent the density of susceptibles, infected asymptomatic and infected symptomatic individuals, respectively. The total population is denoted by *N* and is constant in time. The infected asymptomatic individuals are structured by the age of infection, denoted by $\tau$, i.e. the time that has passed since the individual became infected. The parameters $\beta _1$ and $\beta _2$ are the transmission rates of asymptomatic and symptomatic individuals, respectively, and $\gamma _1$ and $\gamma _2$ are the recovering rates of asymptomatic and symptomatic individuals, respectively. Infected individuals that reach age of infection *T* develop symptoms with probability *p*. Therefore, an infected individual can recover without presenting symptoms if the recovering happens before the age of infection attains *T* or if at age of infection *T* it recovers with probability $1-p$. For more details, see Barril et al. (2021b).

Although in this system the infected individuals are structured by the age of infection, the system can be analysed as if there were no differences between the infected individuals. This is so because all infected individuals have the same properties. Therefore, following the homogeneous population formalism described in the previous section, in order to determine the final infection size and the final symptomatic size, denoted by $\pi$ and $\pi _\text {sym}$, respectively, we must compute the reproduction number associated with the model ($\mathcal {R}_0$) and the probability that an infected individual presents symptoms at some point ($p_\text {sym}$).

The probability $p_\text {sym}$ can be computed noticing that for an infected individual to become symptomatic, it should not recover before age of infection *T*, and then, it should present symptoms, which occurs with probability *p*. Since $\gamma _1(\tau )$ is the recovering rate of asymptomatic individuals with age of infection $\tau$, the probability that an asymptomatic individual does not recover before age of infection *T* is:

$$\begin{aligned} e^{-\int _0^T\gamma _1(\tau )\textrm{d}\tau }, \end{aligned}$$

so that

$$\begin{aligned} p_\text {sym}=e^{-\int _0^T\gamma _1(\tau )\textrm{d}\tau }\,p. \end{aligned}$$

Now we compute $\mathcal {R}_0$, understood as the expected secondary asymptomatic cases produced by an asymptomatic primary case. The expression for $\mathcal {R}_0$ was given but not derived in Barril et al. (2021b). In order to compute it, we follow the formalism developed in Diekmann et al. (1990) (see also Barril et al. 2018, 2021a, b) where $\mathcal {R}_0$ is obtained as the spectral radius of the so-called *next-generation operator*. Specifically we linearize system (23) around the disease-free steady state (*N*, 0, 0) to obtain the following equations for the dynamics of the infected population:

$$\begin{aligned} \begin{array}{l} \partial _t i (t, \tau ) + \partial _{\tau } i (t, \tau ) = - \gamma _1 (\tau ) i (t, \tau ),\\ i (t, 0) = \int _0^T \beta _1 (\tau ) i (t, \tau ) \textrm{d} \tau + \beta _2 J (t), \\ J' (t) = p i (t, T) - \gamma _2 J (t). \end{array} \end{aligned}$$

Then, defining as an infection event the moment in which an individual becomes asymptomatic we can consider the following birth/infection operator

$$\begin{aligned} B:\,\,L^1(0,T)\times \mathbb {R}\longrightarrow & {} \left\langle \delta _0\right\rangle \\ \left( \begin{array}{c} i\\ J \end{array}\right)\longmapsto & {} \left( \int _0^T \beta _1 (\tau ) i (\tau ) \textrm{d} \tau + \beta _2 J\right) \delta _0 \end{aligned}$$

and the mortality/transition operator

$$\begin{aligned} M:\,\,D_M\subset L^1(0,T)\times \mathbb {R}\longrightarrow & {} L^1(0,T)\times \mathbb {R}\\ \left( \begin{array}{c} i\\ J \end{array}\right)\longmapsto & {} \left( \begin{array}{c} i'(\cdot )+\gamma _1(\cdot )i(\cdot )\\ -p\, i(T) + \gamma _2 J \end{array}\right) \end{aligned}$$

with

24 $$\begin{aligned} D_M=\left\{ (i,J) \in W^{1,1}(0,T) \times \mathbb {R}: i(0)=0 \right\} . \end{aligned}$$

The space $\left\langle \delta _0\right\rangle$ is the space generated by a Dirac delta centred at 0. Due to the fact that the range of *B* is not a subspace of its domain, i.e. $\left\langle \delta _0\right\rangle \nsubseteq L^1(0,T)\times \mathbb {R}$, $\mathcal {R}_0$ cannot be defined as the spectral radius of the next-generation operator defined by $BM^{-1}$. However, as shown in Barril et al. (2021a), in the present case $\mathcal {R}_0$ can be computed as:

$$\begin{aligned} \mathcal {R}_0=\lim _{k\rightarrow \infty } \tilde{B} M^{-1}\varphi _k \end{aligned}$$

where $\tilde{B}$ is the functional from $L^1(0,T)\times \mathbb {R}$ to $\mathbb {R}$ defined as

$$\begin{aligned} \tilde{B}\left( \begin{array}{c} i\\ J \end{array}\right) = \int _0^T \beta _1 (\tau ) i (\tau ) \textrm{d} \tau + \beta _2 J \end{aligned}$$

and $\{\varphi _k\}_{k\in \mathbb {N}}\subset L^1(0,T)\times \mathbb {R}$ is any sequence converging to the pair $(\delta _0,0)$, or more specifically, it is any sequence such that

$$\begin{aligned} \lim _{k\rightarrow \infty } M^{-1}\varphi _k = G_M(\cdot ,0) \end{aligned}$$

where $G_M(\cdot ,0)$ is the Green function of the operator *M* associated to the impulse $(\delta _0,0)$. In particular, since $\tilde{B}$ is continuous, then $\mathcal {R}_0$ can be expressed as

$$\begin{aligned} \mathcal {R}_0=\tilde{B}G_M(\cdot ,0). \end{aligned}$$

Therefore, to compute $G_M(\cdot ,0)$, first we determine the preimages of $\varphi _k=(\hat{i}_k, \hat{J}_k)\in L^1(0,T)\times \mathbb {R}$ under the linear operator *M* with domain given in (24). To do that, let us consider

$$\begin{aligned} \left( \begin{array}{c}i_k\\ J_k\end{array}\right) \in D_M\quad \text {such that}\quad \left( \begin{array}{c} i_k\\ J_k\end{array}\right) =M^{-1}\left( \begin{array}{c}\hat{i}_k\\ \hat{J}_k\end{array}\right) , \end{aligned}$$

which implies, by applying *M* to both sides and using that $i_k(0)=0$ (because $(i_k,J_k)\in D_M$),

$$\begin{aligned} \left( \begin{array}{c} i_k'(\cdot )+\gamma _1(\cdot )i_k(\cdot )\\ -p\,i_k(T) + \gamma _2J_k\end{array}\right) =\left( \begin{array}{c}\hat{i}_k\\ \hat{J}_k\end{array}\right) \;\;\Rightarrow \begin{array}{l} i_k(\tau )=\int _0^\tau \hat{i}_k(s)e^{-\int _s^\tau \gamma _1(\sigma )\textrm{d}\sigma }\textrm{d}s, \\ \\ \begin{array}{rcl}J_k&{}=&{}\frac{\hat{J}_k+p\,i_k(T)}{\gamma _2}\\ {} &{}=&{}\left( \hat{J}_k+p\,\int _0^T \hat{i}_k(s)e^{-\int _s^T \gamma _1(\sigma )\textrm{d}\sigma }\textrm{d}s\right) \gamma _2^{-1}.\end{array} \end{array} \end{aligned}$$

With these expressions we finally have, defining $\Gamma _1 (\tau ):= e^{\int _0^{\tau } \gamma _1 (s) \textrm{d} s}$,

$$\begin{aligned} \begin{aligned} G_M(\cdot ,0)&= \lim _{k\rightarrow \infty } M^{-1}\varphi _k=\lim _{k\rightarrow \infty } \left( \begin{array}{c}\int _0^\cdot \hat{i}_k(s)e^{-\int _s^\cdot \gamma _1(\sigma )\textrm{d}\sigma }\textrm{d}s \\ \\ \left( \hat{J}_k+p\,\int _0^T \hat{i}_k(s)e^{-\int _s^T \gamma _1(\sigma )\textrm{d}\sigma }\textrm{d}s\right) \gamma _2^{-1}\end{array}\right) \\&= \left( \begin{array}{c}\int _0^\cdot \delta _0(s)e^{-\int _s^\cdot \gamma _1(\sigma )\textrm{d}\sigma }\textrm{d}s \\ \\ \left( 0+p\,\int _0^T \delta _0(s)e^{-\int _s^T \gamma _1(\sigma )\textrm{d}\sigma }\textrm{d}s\right) \gamma _2^{-1}\end{array}\right) = \left( \begin{array}{c}e^{-\int _0^\cdot \gamma _1(\sigma )\textrm{d}\sigma } \\ \\ p\,e^{-\int _0^T \gamma _1(\sigma )\textrm{d}\sigma }\gamma _2^{-1}\end{array}\right) \\ {}&=\left( \begin{array}{c}\frac{1}{\Gamma _1(\cdot )} \\ \\ \frac{p}{\Gamma _1(T)\gamma _2}\end{array}\right) . \end{aligned} \end{aligned}$$

The reproduction number is then:

$$\begin{aligned} \mathcal {R}_0=\tilde{B}G_M(\cdot ,0) = \int _0^T \frac{\beta _1 (\tau )}{\Gamma _1(\tau )} \textrm{d} \tau + \frac{p\,\beta _2}{\Gamma _1(T)\gamma _2}, \end{aligned}$$

which is meaningful from the biological point of view since the right-hand side of the expression above can be interpreted as the expected secondary infections produced by an infected individual during the asymptomatic phase plus the probability that an infected individual presents symptoms, that is $p_\text {sym}=p/\Gamma _1(T)$, multiplied by the expected secondary infections produced by a symptomatic individual, that is $\beta _2/\gamma _2$.

Once the expressions for $\mathcal {R}_0$ and $p_\text {sym}$ in terms of the parameters of the model are determined, equations (5) and (6) can be used to compute $\pi$ and $\pi _\text {sym}$, that are

$$\begin{aligned} \pi =1-\exp \left( -\pi \left( \int _0^T \frac{\beta _1 (\tau )}{\Gamma _1(\tau )} \textrm{d} \tau + \frac{p\,\beta _2}{\Gamma _1(T)\gamma _2}\right) \right) \qquad \text {and}\qquad \pi _\text {sym}=\frac{p}{\Gamma _1(T)}\pi . \end{aligned}$$

### Remark

Notice that $\pi _\text {sym}$ does not satisfy $\pi _\text {sym}=1-e^{-\tilde{R}_0 \pi _\text {sym}}$ where $\tilde{R}_0$ denotes the secondary symptomatic cases produced by a primary symptomatic individual in a fully susceptible population. This quantity is computed (for the model above) in Barril et al. (2021b) and is given by

$$\begin{aligned} \tilde{R}_0 = \left\{ \begin{array}{ll} \frac{\beta _2}{\gamma _2}\frac{e^{-\Gamma _1(T)}p}{1-\int _0^T \beta _1(\tau ) e^{-\Gamma _1(\tau )}\textrm{d}\tau } &{}\text { if }\int _0^T \beta _1(\tau ) e^{-\Gamma _1(\tau )}\textrm{d}\tau < 1\\ \infty &{} \text { if }\int _0^T \beta _1(\tau ) e^{-\Gamma _1(\tau )}\textrm{d}\tau \ge 1 \end{array} \right. . \end{aligned}$$

This means that formula (5) is not valid for arbitrary definitions of what an “infection event” is, for instance, when considering that the “infection event" occurs when an individual starts presenting symptoms. The reason why (5) fails in this case is that the test individual may become immune without ever entering the symptomatic compartment. Specifically, the first symptomatic individual may cause the test individual to become immune without becoming symptomatic, and in this case the probability that the second symptomatic individual (and the third, the fourth, etc.) causes that the test individual presents symptoms is zero (but the test individual has never presented symptoms!). That is, the probability that a random symptomatic individual causes the symptoms of the test individual depends on the presence of other symptomatic individuals: it is $\tilde{R}_0/N$ if there is only one symptomatic individual throughout the epidemic, but it is going to be smaller than $\tilde{R}_0/N$ if number of accumulated symptomatic individuals is bigger than one. In fact, such a probability decreases with the accumulated number of symptomatic individuals at the end of the epidemics, denoted by $\tilde{K}(N)$, and this prevents us from following the reasoning of section 2.1 since then

$$\begin{aligned} P(u\text { symptomatic})\ne P\left( \text {Bin}\left( \tilde{K}(N),\frac{\tilde{R}_0}{N}\right) \ge 1\right) . \end{aligned}$$

## Model with Individual Heterogeneity (Finite Number of Classes)

Let us consider an epidemic model with individual heterogeneity where the susceptible and infected populations are structured by the discrete variables $x\in \{x_1,\dots ,x_n\}$ and $y\in \{y_1,\dots ,y_n\}$, respectively, and is described by the dynamical equations

25 $$\begin{aligned} \begin{array}{rcl} S_i'(t)&{}=&{}- \frac{1}{N} \left( S_i (t) \sum _{j=1}^n \beta _{ij} I_j (t) + S_i (t)\sum _{j=1}^n \beta ^\text {sym}_{ij} J_j (t) \right) \\ \\ I_i' (t)&{}=&{} \frac{1}{N} \left( S_i (t) \sum _{j=1}^n \beta _{ij} I_j (t) + S_i (t)\sum _{j=1}^n \beta ^\text {sym}_{ij} J_j (t) \right) -p_i I_i(t)-\gamma _i I_i(t)\\ \\ J_i'(t) &{}=&{} p_i(x) I_i (t) - \gamma ^\text {sym}_i J_i (t)\\ \\ \end{array},\qquad \end{aligned}$$

for $$i\in \{1,\dots ,n\}$$.

Here $S_i(t)=S(t,x_i)$ denotes the susceptible population with state $x_i$ at time *t*, whereas $I_i(t)=I(t,y_i)$ and $J_i=J(t,y_i)$ denote, respectively, the asymptomatic and symptomatic populations with state $y_i$ at time *t*. The parameter $p_i=p(y_i)$ denotes the probability per unit of time that an infected individual with state $y_i$ develops symptoms (it corresponds to the rate at which an infected individual presents symptoms), and $\gamma _i=\gamma (y_i)$ and $\gamma ^\text {sym}_i=\gamma ^\text {sym}(y_i)$ the recovery rates of asymptomatic and symptomatic individuals with state $y_i$, respectively. The parameter $\beta _{ij}=\beta (x_i,y_j)$ is the transmission rate between asymptomatic individuals with state $y_j$ and susceptible individuals with state $x_i$, and $\beta ^\text {sym}_{ij}=\beta ^\text {sym}(x_i,y_j)$ stands for the transmission rate between symptomatic individuals with state $y_j$ and susceptible individuals with state $x_i$. Notice that the previous equations imply that after infection, a susceptible of type $x_i$ becomes an infected individual of type $y_i$. Notice also that we are implicitly assuming that infected individuals start by being asymptomatic and only after some time may become symptomatic.

The dynamics of the epidemic is supposed to be sufficiently fast, and therefore, the demographic processes are not considered. Let *N* be the total population at the beginning of the epidemic. There is a continuum of disease-free steady states, which are

$$\begin{aligned} \begin{array}{l} S^0_i=N s_0(x_i)\\ I^0_i=0\\ J^0_i=0\\ \end{array},\qquad \text {for}\quad i\in \{1,\dots ,n\} \end{aligned}$$

with $s_0(x_i)\ge 0$ for all $x_i$ and

$$\begin{aligned} \sum _{i=1}^n s_0(x_i) = 1. \end{aligned}$$

Linearizing around one of these disease-free steady states, we obtain the following equations for the dynamics of the infected population:

$$\begin{aligned} \begin{array}{rcl} I_i' (t)&{}=&{} \left( s_0(x_i) \sum _{j=1}^n \beta _{ij} I_j (t) + s_0(x_i)\sum _{j=1}^n \beta ^\text {sym}_{ij} J_j (t) \right) -p_i I_i(t)-\gamma _i I_i(t)\\ \\ J_i'(t) &{}=&{} p_i I_i (t) - \gamma ^\text {sym}_i J_i (t) \end{array}, \end{aligned}$$

for $$i\in \{1,\dots ,n\}$$.

Clearly, the state space of the linearized system is $\mathbb {R}^n\times \mathbb {R}^n$. Let us use a basis of this space so that the states are written as pairs of *n*-tuples:

$$\begin{aligned} ((I_1,\dots ,I_n)^\top ,(J_1,\dots ,J_n)^\top ). \end{aligned}$$

Denoting by $\{e_1,\dots ,e_n\}$ the canonical basis of $\mathbb {R}^n$, the basis of the state space $\mathbb {R}^n\times \mathbb {R}^n$ is then:

$$\begin{aligned} \{(e_1,0)^\top ,\dots ,(e_n,0)^\top ,(0,e_1)^\top ,\dots ,(0,e_n)^\top \}. \end{aligned}$$

Defining as an infection event the moment in which an individual becomes asymptomatic we can consider the following birth/infection operator *B* and mortality/transition operator *M*.

$$\begin{aligned} \begin{array}{ccc} B:=\left( \begin{array}{cc} \text {D}(s_0)\beta &{} \text {D}(s_0)\beta ^\text {sym}\\ \\ 0 &{} 0 \end{array}\right) &{}, &{} M:=\left( \begin{array}{cc}\text {D}(p+\gamma ) &{} 0 \\ \\ -\text {D}(p) &{} \text {D}(\gamma ^\text {sym})\end{array}\right) , \end{array} \end{aligned}$$

where $\text {D}(v)$ denotes a diagonal matrix whose diagonal entries are given by vector *v*, where $s_0$, $\gamma$, $\gamma ^\text {sym}$ and *p* denote vectors whose *i*th entries are, respectively, $s_0(x_i)$, $\gamma _i$, $\gamma ^\text {sym}_i$ and $p_i$, and where $\beta$ and $\beta ^\text {sym}$ are matrices whose entries (row *i*, column *j*) are $\beta _{ij}$ and $\beta ^\text {sym}_{ij}$ respectively.

With these two operators, we obtain the so-called *next generation operator* (Diekmann et al. 1990; Inaba 2017; Barril et al. 2021b)

$$\begin{aligned} \begin{aligned} BM^{-1}&=B\left( \begin{array}{cc}\text {D}\left( \frac{1}{p+\gamma }\right) &{} 0 \\ \\ \text {D}\left( \frac{p}{(p+\gamma )(\gamma ^\text {sym})}\right) &{} \text {D}\left( \frac{1}{\gamma ^\text {sym}}\right) \end{array}\right) \\ \\&=\left( \begin{array}{cc} \text {D}(s_0)\beta \text {D}\left( \frac{1}{p+\gamma }\right) +\text {D}(s_0)\beta ^\text {sym}\text {D}\left( \frac{p}{(p+\gamma )\gamma ^\text {sym}}\right) &{} \text {D}(s_0)\beta ^\text {sym}\text {D}\left( \frac{1}{\gamma ^\text {sym}}\right) \\ \\ 0 &{} 0 \end{array}\right) \end{aligned} \end{aligned}$$

where, with slight abuse of notation, for any vector *v* with positive components, $\frac{1}{v}$ denotes the vector whose components are the inverse of the components of *v*.

With the *next-generation operator*, we can compute the number of secondary infections an infected individual of type $y_j$ causes to susceptibles of type $x_i$. It is enough to apply this operator to the *j*th basis vector of $\mathbb {R}^n\times \mathbb {R}^n$, namely $(e_j,0)$ (this represents the situation in which there is only one infected asymptomatic individual of type $y_j$, i.e. $I_j=1$ and $I_i=0$ for all $i\ne j$ and $J_i=0$ for all *i*). The *i*th index of the first half of the resultant vector $BM^{-1}(e_j,0)$ gives the number of secondary cases of type $y_i$ produced by the primary infected individual of type $y_j$ (notice that $BM^{-1}(e_j,0)$ has 2*n* components, but that the second half of the vector is full of zeros due to the assumption that new infected individuals are always asymptomatic). Since all infected individuals of type $y_i$ were susceptible of type $x_i$, we conclude that the number of secondary infections an infected individual of type $y_j$ causes to susceptibles of type $x_i$ is the scalar product of $(e_i,0)^\top$ by $BM^{-1}(e_j,0)^\top$, i.e.,

$$\begin{aligned} \begin{aligned} \mathcal {R}_0(x_i,y_j)&=\left\langle (e_i,0)^\top ,BM^{-1}(e_j,0)^\top \right\rangle \\ \\ {}&=\left\langle e_i,\left( \text {D}(s_0)\beta \text {D}\left( \frac{1}{p+\gamma }\right) +\text {D}(s_0)\beta ^\text {sym}\text {D}\left( \frac{p}{(p+\gamma )\gamma ^\text {sym}}\right) \right) e_j\right\rangle \\ \\ {}&=s_0(x_i)\left( \frac{\beta _{ij}}{p_j+\gamma _j}+\frac{p_j\,\beta ^\text {sym}_{ij}}{(p_j+\gamma _j)\gamma ^\text {sym}}\right) \end{aligned} \end{aligned}$$

The final infection size of the different classes, i.e. $\pmb {\pi }=(\pi (y_1),\dots ,\pi (y_n))$ is a fixed point of equation (12), that is

26 $$\begin{aligned} \pmb {\pi }=F(\pmb {\pi }) \end{aligned}$$

with $F:\mathbb {R}^n\rightarrow \mathbb {R}^n$ and the *i*th component function of *F* being defined, for a vector $v=(v_1,\dots ,v_n)$, as

27 $$\begin{aligned} F_i(v) = \left( 1-e^{-\sum _{j=1}^n \left( \frac{\beta _{ij}}{p_j+\gamma _j}+\frac{p_j\,\beta ^\text {sym}_{ij}}{(p_j+\gamma _j)\gamma ^\text {sym}}\right) v_j}\right) s_0(x_i). \end{aligned}$$

Once we know the final infection size of each class, we can compute the final symptomatic size. To do this, we have to determine the probability that an infected individual of type $y_i$ presents symptoms. Since the recovery rate of this individual is $\gamma _i$ and the rate of presenting symptoms is $p_i$, the probability that this individual presents symptoms is

$$\begin{aligned} p_\text {sym}(y_i)=\frac{p_i}{p_i+\gamma _i}. \end{aligned}$$

Consequently, the final symptomatic size of infected individuals of type $y_i$ (that coincides with the final symptomatic size of susceptible individuals of type $x_i$ because the classes of susceptible and infected individuals coincide, i.e., $\pi _\text {sym}(y_i)=\pi ^\text {sym}_s(x_i)$) is

$$\begin{aligned} \pi _\text {sym}(y_i)=\pi (y_i)p_\text {sym}(y_i)=\pi (y_i)\frac{p_i}{p_i+\gamma _i}. \end{aligned}$$

**Fig. 1:**
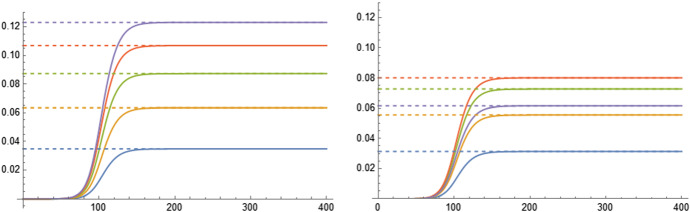
Comparison between the theoretical result (dashed lines) and the numerical result obtained by integrating system (25) of Sect. 4 (continuous lines). The left plot shows the infected size of a system with 5 different classes with respect to time (the dashed lines represent $\pmb {\pi }$), and the right plot shows the symptomatic size of the same system and the same classes (the dashed lines represent $\pmb {\pi }_\text {sym}$). Notice that for this particular example the class that has the largest final infected size (the class represented in purple) is not the class that has the largest final symptomatic size (the class represented in red). Adding the different dashed lines we would obtain the total final infected size $\pi$ (in the left) and the total final symptomatic size $\pi _\text {sym}$ (in the right). The parameters of the simulation are (following the notation in the main text): $n=5$, $s_0(x_i)=1/n$, $\beta _{ij}=ij/n^2$, $\beta ^{\text {sym}}_{ij}=0$, $\gamma _i=0.1$, $\gamma ^\text {sym}_i=0.2$ and $p_i=1.1-i/n$ for $i,j\in \{1,\dots ,n\}$

Once $\pmb {\pi }$ and $\pmb {\pi }_\text {sym}=(\pi _\text {sym}(y_1),\dots ,\pi _\text {sym}(y_n))$ are determined, the final infected and symptomatic sizes can be computed using (15) and (22). In Fig. 1, we compare the theoretical and numerical results of a specific example of system (25).

## Model with Individual Heterogeneity (Continuous Trait)

Let us consider the continuous extension of the model presented in the previous section. Specifically let us assume that individual heterogeneity is expressed by a continuous variable *x* taking values in $\Omega =[0,1]$, and let us consider the equations

$$\begin{aligned} \begin{array}{rcl} \partial _t s(t,x)&{}=&{}\displaystyle - \frac{1}{N} \left( s(t,x) \int _{\Omega } \beta _1 (x,y) i (t, y) \textrm{d}y + s(t,x)\int _{\Omega } \beta _2 (x,y) j (t,y)\textrm{d}y \right) \\ \\ \partial _t i (t, x)&{}=&{} \displaystyle \frac{1}{N} \left( s (t,x) \int _{\Omega } \beta _1 (x,y) i (t, y) \textrm{d}y + s (t,x)\int _{\Omega } \beta _2 (x,y) j (t,y)\textrm{d}y \right) \\ \\ {} &{} &{} -p(x)i(t,x)-\gamma _1(x)i(t,x)\\ \\ \partial _t j(t,x) &{}=&{} p(x) i (t, x) - \gamma _2(x) j (t,x)\\ \end{array} \end{aligned}$$

where *s*(*t*, *x*) denotes the susceptible, *i*(*t*, *x*) the asymptomatic and *j*(*t*, *x*) the symptomatic population density with state *x* at time *t*. As a consequence, the phase space of the above system is set to be $L^1(0,1)^3$. Here *p*(*x*) denotes the probability of developing symptoms, and $\gamma _1(x)$ and $\gamma _2(x)$ the recovery rates of asymptomatic and symptomatic individuals, respectively. $\beta _1(x,y)$ is the transmission rate between asymptomatic individuals with state *y* and susceptible individuals with state *x*, and $\beta _2(x,y)$ stands for the transmission rate between symptomatic individuals with state *y* and susceptible individuals with state *x*.

As before, the dynamics of the epidemic is supposed to be sufficiently fast, and therefore, the demographic processes are not considered and *N* is the total population at the beginning of the epidemic.

Linearizing around a disease-free steady state $(N s_0(x),0,0)$, with $\int _0^1 s_0(x)\textrm{d}x=1$, we obtain the following equations for the dynamics of the infected population:

$$\begin{aligned} \begin{array}{rcl} \partial _t i (t, x)&{}=&{} \displaystyle s_0 (x) \int _{\Omega } \beta _1 (x,y) i (t, y) \textrm{d}y + s_0(x)\int _{\Omega } \beta _2 (x,y) j (t,y)\textrm{d}y \\ \\ {} &{} &{} -p(x)i(t,x)-\gamma _1(x)i(t,x),\\ \\ \partial _t j(t,x) &{}=&{} p(x) i (t, x) - \gamma _2(x) j (t,x) \end{array} \end{aligned}$$

The birth/infection operator *B* and mortality/transition operator *M* (unlike the example of Sect. 3 here both operators are bounded, i.e. they are defined in all $L^1(0,1)^2$) are

$$\begin{aligned} B:= & {} \left( \begin{array}{cc} s_0(\hat{x}) \int _{\Omega } \beta _1 (\hat{x},\hat{y}) \cdot \textrm{d}\hat{y} &{} s_0(\hat{x}) \int _{\Omega } \beta _2 (\hat{x},\hat{y}) \cdot \textrm{d}\hat{y}\\ \\ 0 &{} 0 \end{array}\right) , \\ M:= & {} \left( \begin{array}{cc}p(\hat{x})+\gamma _1(\hat{x}) &{} 0 \\ \\ -p(\hat{x}) &{} \gamma _2(\hat{x})\end{array}\right) , \end{aligned}$$

and the *next-generation operator* is thenHere $\hat{x}$ and $\hat{y}$ have been used (instead of *x* and *y*) in order to avoid a notational collision in formula (29) below.

With an analogous argument as in the previous section, we obtain the “continuous” version of equations (26) and (27). Specifically, the final infection density is a solution of the functional equation

28 $$\begin{aligned} F(\tilde{\pi })=\tilde{\pi } \end{aligned}$$

with $F:L^1(0,1)\rightarrow L^1(0,1)$ defined as (recall (18))

$$\begin{aligned} \begin{aligned} F(\tilde{\pi })(x)&=\left( 1-e^{-\int _{\Omega } \frac{\mathcal {R}_0(x,y)}{s_0(x)}\tilde{\pi }(y)\textrm{d}y}\right) s_0(x) \\ {}&= \left( 1-e^{-\int _{\Omega _Y} \left( \frac{\beta _1 (x,y)}{p(y)+\gamma _1(y)} +\frac{\beta _2 (x,y)p(y)}{(p(y)+\gamma _1(y))\gamma _2(y)}\right) \tilde{\pi }(y)\textrm{d}y}\right) s_0(x) \end{aligned} \end{aligned}$$

since $\mathcal {R}_0(x,y)$ is given by

29 $$\begin{aligned} \begin{aligned} \mathcal {R}_0(x,y)&=\left\langle \left( \begin{array}{c} \delta _x \\ 0 \end{array}\right) ,BM^{-1}\left( \begin{array}{c} \delta _y \\ 0 \end{array}\right) \right\rangle \\ {}&= s_0(x) \left( \frac{\beta _1 (x,y)}{p(y)+\gamma _1(y)} +\frac{\beta _2 (x,y)p(y)}{(p(y)+\gamma _1(y))\gamma _2(y)}\right) . \end{aligned} \end{aligned}$$

Finally, since the probability that an infected individual of class *y* presents symptoms is:

$$\begin{aligned} p_\text {sym}(y)=\frac{p(y)}{p(y)+\gamma _1(y)}, \end{aligned}$$

the final density of symptomatic cases, structured by the variable *y*, is

$$\begin{aligned} \tilde{\pi }_\text {sym}(y)=\tilde{\pi }(y)p_\text {sym}(y)=\tilde{\pi }(y)\frac{p(y)}{p(y)+\gamma _1(y)}. \end{aligned}$$

and the total number of symptomatic cases at the end of the epidemic is given by (whenever the solution $\tilde{\pi }$ of (28) can be obtained) as

$$\begin{aligned} \pi _\text {sym}=\int _\Omega \tilde{\pi }(y)\frac{p(y)}{p(y)+\gamma _1(y)} \textrm{d}y. \end{aligned}$$

## Conclusion

In this article, we used the probability for a test individual to become infected and develop symptoms to compute the final number of symptomatic cases that the epidemic will cause. The obtained equations relate the final symptomatic cases with the reproduction number of the epidemics and the probability/rates at which infected individuals present symptoms. The equations are, therefore, natural generalizations to the well-known final infection size equations.

It has been discussed elsewhere (see, for instance, Cushing and Diekmann 2016) that basic reproduction numbers depend on how an “infection event” is defined. From a biological point of view, it makes sense to consider that individuals become infected as soon as the infectious agents start to proliferate within them (a *biological infection event*). However, in practical situations most infected individuals are not reported in the course of an epidemic unless they present symptoms at some time, so that in some sense the onset of symptoms is what defines the “infection event” in epidemiological data (an *epidemiological infection event*). In this article, $\mathcal {R}_0$ is always associated with the *biological infection event* definition, which explains why the probability of presenting symptoms appears as an independent element in the final symptomatic cases equation. A natural question (and what motivated part of this work) is what happens when adopting the *epidemiological infection event* definition. More precisely, taking into account that $\pi =1-e^{-\mathcal {R}_0\pi }$ is an equation for the final infection size in the homogeneous case (Sect. 2.1), does an analogous formula for the final symptomatic cases hold when the reproduction number is computed according to the *epidemiological infection event* definition? That is, denoting by $\tilde{\mathcal {R}}_0$ such an alternative reproduction number (which gives the secondary symptomatic cases produced by a symptomatic individual), then does $\pi _\text {sym}=1-e^{-\tilde{\mathcal {R}}_0\pi _\text {sym}}$ hold? The answer is no (as shown in the remark at the end of section 3). The reason is that the test individual argument used to deduce (5) cannot be applied in the same way considering only the symptomatic population and $\tilde{\mathcal {R}}_0$. The difference in this setting is that the test individual may gain immunity (i.e. recover) at some point without having been part of the symptomatic population (while in the reasoning leading to (5) the test individual stays susceptible until it, eventually, becomes part of the infected population).

This observation has important implications on how the final infection size is computed with the available information. Indeed, if Eq. (5) is used plugging the reproduction number associated with symptomatic individuals, the result would neither be the final size of symptomatic cases nor the final infection size.

Although adding realism in terms of population structure, the formalism presented here still relies on important simplifying assumptions, such as the time-independence of the susceptibility level discussed in section 2.1. Recent work has analysed the final size for situations where there is a permanent reduction in mixing intensity (and hence a reduction in the susceptibility level) that depends on the epidemic progression (Gog and Hollingsworth 2021). Although the test individual trick does not seem to be generalizable in a straightforward way in time-dependent scenarios, it is worth studying if the trick can be extended at least to the simple model considered in Gog and Hollingsworth (2021), for which final infection size formulas do exist. If this were the case, maybe these formulas could be generalized to heterogeneous population (with possibly asymptomatic individuals) proceeding as we do in this paper.

## Appendix A Proof of Theorem 1

To prove Theorem 1, we follow the ideas in Magal et al. (2018, 2016), where a similar problem is addressed. The results stem from the theory of monotone discrete dynamical systems (see section 5 in Hirsch and Smith 2005). First we define the relation $\le$ between elements of $\mathbb {R}^m$ as

$$\begin{aligned} u \le v \qquad \text {if}\qquad u_i\le v_i\quad \forall i\in \{1,\dots ,m\}, \end{aligned}$$

and the interval [*u*, *v*] as the set of all $w\in \mathbb {R}^m$ satisfying $u\le w \le v$. In addition, we consider the three following general results.

### Proposition 2

Let $F:\mathbb {R}^m\rightarrow \mathbb {R}^m$ be component-wise increasing, i.e. such that $F(u)\le F(v)$ if $u\le v$. Let $u_0$ and $v_0$ such that $F^l(u_0)\rightarrow u_\infty$ and $F^l(v_0)\rightarrow v_\infty$ as $l\rightarrow \infty$. Let $w\in [u_0,v_0]$. If $\bar{w}$ is an accumulation point of the orbit of *w* by *F*, then $\bar{w}\in [u_\infty ,v_\infty ]$.

### Proof

Since $u_0\le w\le v_0$, by the monotony of *F* we have $F(u_0)\le F(w)\le F(v_0)$, and applying this argument inductively it follows $F^l(u_0)\le F^l(w)\le F^l(v_0)$ for all $l\in \mathbb {N}$. Now let $\bar{w}$ be an accumulation point of the orbit of *w* by *F*, i.e. there exist an unbounded subset of indices $\{l_k\}_{k\in \mathbb {N}}$ such that

$$\begin{aligned} \lim _{k\rightarrow \infty } F^{l_k}(w)=\bar{w}, \end{aligned}$$

which implies

$$\begin{aligned} u_\infty = \lim _{k\rightarrow \infty } F^{l_k}(u_0) \le \bar{w} \le \lim _{k\rightarrow \infty } F^{l_k}(v_0) = v_\infty . \end{aligned}$$

$$\square $$

Let *F* be as in Proposition 2 and let *u* and *v* be fixed points of *F*. A doubly infinite sequence $\{x_n\}_{n\in \mathbb {Z}}$ in $\mathbb {R}^n$ is called an *entire orbit from **u** to **v* if

$$\begin{aligned} x_{n+1}=F(x_n),\quad \lim _{n\rightarrow -\infty } x_n = u,\quad \quad \lim _{n\rightarrow \infty } x_n = v. \end{aligned}$$

### Proposition 3

(Theorem 5.11 of Hirsch and Smith 2005) Let *F* be as in Proposition 2 and continuous. Let *u* and *v*, with $u\le v$, be two fixed points of *F*. Then at least one of the following holds:

- there is a third fixed point $\bar{w}$ such that $u\le \bar{w} \le v$;
- there exists an entire orbit from *u* to *v* that is increasing;
- there exists an entire orbit from *v* to *u* that is decreasing.

### Proposition 4

(Corollary 5.12 of Hirsch and Smith 2005) Let *F*, *u* and *v* as in Proposition 3. If both *u* and *v* are locally asymptotically stable, then *F* has a third fixed point $\bar{w}\in [u,v]$, which is not locally asymptotically stable.

Although the dynamical behaviour of the third fixed point $\bar{w}$ is not stated in Corollary 5.12 of Hirsch and Smith (2005), notice that at least one fixed point in [*u*, *v*] will not be locally asymptotically stable. To see this, let *A* be the set of all fixed points of *F* in [*u*, *v*] which are locally asymptotically stable. Since [*u*, *v*] is compact, *A* is necessarily finite. Otherwise there would exist an accumulation point in [*u*, *v*] of locally asymptotically stable fixed points, which would imply that such point would converge to infinitely many different points, which is impossible. Choose $v'\in A$ such that the only elements of *A* in $[u,v']$ are *u* and $v'$. Then, Corollary 5.12 of Hirsch and Smith (2005) ensures the existence of a third fixed point $\bar{w}\in [u,v']\subset [u,v]$, and $\bar{w}$ is not locally asymptotically stable because $\bar{w}\not \in A$ by construction.

Before proceeding further, let us see how $\mathcal {R}_0$ is related to the mapping given by *F* in (14). Recall that $\mathcal {R}_0$ can be defined as the spectral radius of the next-generation operator *G*. This operator, which is a matrix when there are only finitely many classes of infected individuals, gives the distribution of secondary infections as a function of the distribution of primary infected individuals. In particular, if $e_i$ denotes the *i*th canonical vector, the *j*th entry of the image vector $Ge_i$ (which coincides with the entry (*j*, *i*) of the matrix *G*, i.e. $G_{j,i}$) gives the expected number of secondary infected individuals of class $y_j$ that a primary infected individual of class $y_i$ produces. Taking this observation into account, it is easy to construct the matrix *G* using the values $\mathcal {R}_0(x_k,y_i)$ and $p_{x_k\rightarrow y_j}$. Indeed, since $\mathcal {R}_0(x_k,y_i)$ gives the expected number of susceptible individuals of class *k* infected by a primary infected individual of class *i* and $p_{x_k\rightarrow y_j}$ gives the probability that when a susceptible of class *k* is infected it becomes an infected individual of class *j*, it turns out that

A1 $$\begin{aligned} G_{j,i}=\sum _{k=1}^n \mathcal {R}_0(x_k,y_i)p_{x_k\rightarrow y_j}. \end{aligned}$$

We conclude, therefore, that $\mathcal {R}_0=\rho (G)$ where *G* is the matrix whose entries are defined in (A1).

On the other hand, notice that 0 is a fixed point of *F*. It is well known that the local behaviour of 0 is determined by the spectral radius of *DF*(0) (it is locally asymptomatically stable if $\rho (DF(0))<1$ and it is unstable if $\rho (DF(0))>1$). A quick computation shows that $DF(0)=G$, which implies that 0 is locally asymptomatically stable if $\mathcal {R}_0<1$ whereas it is unstable if $\mathcal {R}_0>1$. To prove that 0 is in fact a global attractor of the positive cone when $\mathcal {R}_0<1$, we need the following results:

### Proposition 5

Let *F* be defined in (14). Then, *F* is component-wise increasing, i.e. $F(u)\le F(v)$ if $u\le v$.

### Proof

Notice that $\partial _i F_j(w)\ge 0$ for all $w\in \mathbb {R}^m$ and $i,j\in \{1,\dots ,m\}$. $\square$

### Proposition 6

Let *F* be defined in (14). Let $u,v\in \mathbb {R}^m$. If $0\le u\le v$, then $\rho (DF(u))\ge \rho (DF(v))$.

### Proof

First notice that the entry (*j*, *i*) of *DF*(*u*), i.e. $\partial _i F_j(u)$, is smaller than the entry (*j*, *i*) of *DF*(*v*), i.e. $\partial _i F_j(v)$. This follows from the fact that $\partial _{ki} F_j(w)\le 0$ for all $i,j,k\in \{1,\dots ,m\}$ and $w\in \mathbb {R}^m$. Moreover, all entries of *DF*(*u*) and *DF*(*v*) are nonnegative. Therefore, $\Vert DF(u)^l\Vert \ge \Vert DF(v)^l\Vert$ for all $l\in \mathbb {N}$, and applying the Gelfand’s formula we finally conclude

$$\begin{aligned} \rho (DF(u))=\lim _{l\rightarrow \infty }\Vert DF(u)^l\Vert ^\frac{1}{l}\ge \lim _{l\rightarrow \infty }\Vert DF(v)^l\Vert ^\frac{1}{l}=\rho (DF(v)). \end{aligned}$$

$$\square $$

### Proposition 7

Let *F* be defined in (14). Define $v^0\in \mathbb {R}^m$ as

$$\begin{aligned} v^0_i=\sum _{k=1}^n s_0(x_k) p_{x_k\rightarrow y_i}\qquad \forall i\in \{1,\dots ,m\}. \end{aligned}$$

Then,

i. the orbit of $v^0$ converges to a fixed point of *F*, i.e.
  $$\begin{aligned} \lim _{l\rightarrow \infty } F^l(v^0)=\bar{v}\quad \text { and }\quad F(\bar{v})=\bar{v}, \end{aligned}$$
ii. for all $w\ge \bar{v}$, $\lim _{l\rightarrow \infty } F^l(w)=\bar{v}$.

### Proof

To prove i. notice that $F^{l+1}(v^0)\le F^l(v^0)$ for all $l\in \mathbb {N}$, so that the sequence $\{F^l(v^0)\}_{l\in \mathbb {N}}$ is decreasing component-wise. Since the orbit of $v^0$ is bounded (notice that $0\le F^l(v_0)\le v_0$ for all $l\in \mathbb {N}$), it follows that the sequence $\{F^l(v^0)\}_{l\in \mathbb {N}}$ converges to a point $\bar{v}\in [0,v^0]\subset \mathbb {R}^m$. Since *F* is continuous, $\bar{v}$ is a fixed point of *F*. To prove ii., notice that for all $w\ge v_0$ one has $F(w)\le v_0$. Then, since *F* is component-wise increasing and $v_0\ge \bar{v}$ and $\lim _{l\rightarrow \infty } F^l(v_0)=\bar{v}= \lim _{l\rightarrow \infty } F^l(\bar{v})$, by applying Proposition 2 it follows that the accumulation points of $\{F^l(w)\}_{l\in \mathbb {N}}$ belong to $[\bar{v},\bar{v}]=\{\bar{v}\}$, which implies that $\{F^l(w)\}_{l\in \mathbb {N}}$ converges to $\bar{v}$. $\square$

As a corollary of these results, it follows that:

### Proposition 8

If $\mathcal {R}_0<1$ then $\lim _{l\rightarrow \infty } F^l(w)=0$ for all $w\in \mathbb {R}_+^m$, with $\mathbb {R}_+:=(0,\infty )$.

### Proof

This is shown by noticing that in this case the $\bar{v}$ of Proposition 7 has to be necessarily 0. Indeed, if $\bar{v}>0$, then by Proposition 4 we have that there exists $\bar{w}\in [0,\bar{v}]$ which is not locally asymptotically stable, but by Proposition 6 it follows $1>\mathcal {R}_0=\rho (DF(0))\ge \rho (DF(\bar{w}))$, which contradicts this fact. $\square$

### Proposition 9

If $\mathcal {R}_0>1$, then $\lim _{l\rightarrow \infty } F^l(w)=\bar{v}\ne 0$ for all $w>\bar{v}$. Moreover, if 0 and $\bar{v}$ are the only fixed points of *F* in the positive cone of $\mathbb {R}^m$, then $\lim _{l\rightarrow \infty } F^l(w)=\bar{v}\ne 0$ for all $w\in \mathbb {R}^m_+$

### Proof

Since the basin of attraction of $\bar{v}$ contains an open set (namely all points $w\in \mathbb {R}^m$ satisfying $w\ge \bar{v}$), necessarily $\rho (DF(\bar{v}))\le 1$. Therefore, since $\mathcal {R}_0=\rho (DF(0))>1$, it follows that $\bar{v}\ne 0$. Applying Proposition 7, we conclude the first part of the statement.

In order to show that the interior of the positive cone of $\mathbb {R}^m$ belongs to the basin of attraction of $\bar{v}$ when 0 and $\bar{v}$ are the only fixed points of *F* in the positive cone, first notice that by Proposition 3 there is an entire orbit from 0 to $\bar{v}$ (since the existence of an entire orbit from $\bar{v}$ to 0 would imply $\rho (DF(0))\le 1$). Therefore, for all $w\in \mathbb {R}_+^m$ we can take $w_-\in (0,\bar{v}]$ from that entire orbit and $w_+\ge \bar{v}$ such that $w\in [w_-,w_+]$. Then, since $\lim _{l\rightarrow \infty } F^l(w_-)=\bar{v}=\lim _{l\rightarrow \infty } F^l(w_+)$, by Proposition 2 we conclude

$$\begin{aligned} \lim _{l\rightarrow \infty } F^l(w)\in [\bar{v},\bar{v}]=\{\bar{v}\}. \end{aligned}$$

$$\square $$
